# Active Holistic Surveillance: The Nutritional Aspect of Delayed Intervention in Prostate Cancer

**DOI:** 10.1155/2016/2917065

**Published:** 2016-05-05

**Authors:** Courtney J. Berg, David J. Habibian, Aaron E. Katz, Kaitlin E. Kosinski, Anthony T. Corcoran, Andrew S. Fontes

**Affiliations:** Department of Urology, Winthrop University Hospital, Mineola, NY 11501, USA

## Abstract

*Purpose*. Active surveillance is an emergent strategy for management of indolent prostate cancer. Our institution's watchful waiting protocol, Active Holistic Surveillance (AHS), implements close monitoring for disease progression along with various chemopreventive agents and attempts to reduce unnecessary biopsies. Our objective is to report on the treatment rates of men on our AHS protocol as well as determine reasons for progression.* Materials/Methods*. Low risk and low-intermediate risk patients were enrolled in AHS at Winthrop University Hospital between February 2002 and August 2015. Our IRB-approved study analyzed survival rate, discontinuation rates, and definitive treatments for patients in our AHS cohort.* Results*. 235 patients met inclusion criteria. Median age and follow-up for the cohort were 66 (44–88) years and 42 (3–166) months, respectively. The overall survival for the cohort was 99.6% and the disease specific survival was 100%. A total of 27 (11.5%) patients discontinued AHS.* Conclusion*. The incorporation of chemopreventive agents in our AHS protocol has allowed patients to prolong definitive treatment for many years. Longer follow-up and additional studies are necessary to further validate the effectiveness of AHS.

## 1. Introduction

Prostate cancer is the second most commonly diagnosed cancer amongst American men [1]. In combination with an aging population, the development of the PSA screening test resulting in early detection has driven the increase in prostate cancer incidence. Nevertheless, treatment of low risk prostate cancer remains unclear. As of July 2008, Cancer of the Prostate Strategic Urologic Research Endeavor (CaPSURE) reported that 95% of prostate cancer patients were receiving definitive treatment, even with most patients having low-grade disease [2]. Definitive treatment causes several side effects for patients, including incontinence, erectile dysfunction, and bowel complications [3]. However, many of these early detected cancers tend to be more indolent than life threatening, and often treatment of this nonlethal disease represents overtreatment. Clinicians thus found it necessary to develop an approach that embraced the concept of watchful waiting for low risk patients to prolong and possibly avoid definitive treatment [4].

This approach, known as active surveillance, is an emerging strategy for management of favorable-risk, localized prostate cancer. The active surveillance regimens that are commonly used involve closely monitoring low risk prostate cancer patients for any sign of disease progression with serial PSA tests, biopsies, and/or MRIs. The goal of any active surveillance program is to try and avoid or delay the side effects of treatment but also to maintain the option of delayed intervention.

At our institution, we have a large cohort of patients on an active surveillance protocol which uses a novel approach of diet and supplements. The protocol, called Active Holistic Surveillance (AHS), involves regular follow-up exams with PSA testing, review of symptoms, digital rectal examinations, and annual MRIs. The protocol does not have routine subsequent prostate biopsies unless there is a rapid rise in PSA or a change in MRI and/or DRE. This serves to decrease the side effects and discomfort of biopsy while attempting to still capture cancer progression before the window of cure is closed.

Epidemiological research has demonstrated that nutrition and lifestyle factors play a pivotal role in the initiation and progression of prostate cancer. However, chemopreventive agents such as nutrients, herbs, and dietary factors have been shown to reduce the aggressiveness of the disease. Implementing these chemopreventive agents into a treatment regimen may prevent progression and occurrence of prostate cancer. In addition to management of prostate cancer, these chemopreventive agents provide defense against various other malignancies and promote the overall health of the patient [5, 6].

This study is looking to determine the definitive treatment rates of the patients on our institution's AHS protocol and look at the reasons for progression. In addition, we also compare our rates of conversion to definitive therapy to other large series in the literature.

*Diet's Role in Prostate Cancer Progression*

*Meat*. The correlation between increased red meat consumption and increased risk for prostate cancer has been extensively studied. An internationally conducted epidemiological study revealed a positive correlation between prostate cancer death and fat consumption, specifically in fats obtained from meats and dairy [7, 16]. While the exact reason for the association with red meat and prostate cancer is unknown, studies suggest grilling or barbecuing meats at high temperatures can form heterocyclic amines and polycyclic aromatic hydrocarbons, both of which have carcinogenic properties in animal models [6].

*Dairy Products*. There is limited data on the relationship between dairy intake and prostate cancer. However, a high intake of saturated and trans fat often found in dairy products is positively correlated with incidence and mortality from prostate cancer [27]. One epidemiological study of 41 countries found milk to be the food most closely related to prostate cancer incidence [28]. While the exact mechanism by which fat induces tumorigenesis is not yet known, possible explanations include fat's effects on serum androgen levels which affect tumor growth. In addition, dairy products contain a significant amount of calcium. In high doses, calcium inhibits the formation of 1,25-dihydroxyvitamin D3, the active form of vitamin D which inhibits the proliferation of cancerous prostatic cells. 

*Soy*. Epidemiological studies report a markedly lower incidence of prostate cancer in Asian countries, which may be attributable to the prevalence of soy based foods in a traditionally Asian diet [6]. Soy products contain numerous phytoestrogens. However, the predominant phytoestrogen comprising soy is genistein. In rat models, genistein has been proven to inhibit the growth and metastasis of prostatic cell carcinomas [29]. In recent years, the incidence rate of prostate cancer in Asian countries has risen, which may be due to the adaptation of western diets into the Asian lifestyle.

*Vegetables*. Studies comparing correlations between food groups and cancer mortality rates found a negative correlation between prostate cancer mortality and the consumption of vegetables [7, 8]. Vegetables are densely packed with antioxidants, which neutralize the effect of heterocyclic amines found in meats [6]. Cruciferous vegetables, which include broccoli, spinach, kale, and cauliflower, also contain glucosinolates. When digested, glucosinolates release numerous phytochemicals which induce the apoptosis of prostate cancer cells and prohibit these cells from proliferating. In addition, glucosinolates function to protect somatic DNA from carcinogenic damage.

*Green Tea*. Green tea contains epigallocatechin gallate (EGCG), an extensively studied catechin known for its anticarcinogenic and antioxidant properties. Like those found in vegetables, EGCG removes carcinogenic free radicals from the body. In addition, green tea exhibits anti-inflammatory properties, contributing to its value as a chemopreventive agent. Numerous cell culture model studies and animal studies have shown green tea's ability to attenuate development, progression, and metastasis of prostate cancer by inducing apoptosis and cell-growth inhibition [30]. Green tea's chemopreventive properties may be attributed to the low incidence of prostate cancer in Japanese and Chinese populations [31].

## 2. Methods

### 2.1. Cohort Enrollment

Between February of 2002 and August 2015, patients with low risk and low-intermediate risk prostate cancer have been enrolled in AHS at Winthrop University Hospital. This study has been approved by the Institutional Review Board.

Criteria for enrollment into AHS include the following: Gleason 6 (3 + 3), low volume Gleason 7 (3 + 4), nonpalpable disease on exam, stage T1 or T2, and no evidence of extracapsular extension on mpMRI. Exceptions were made for men with Gleason 7 (4 + 3) depending on significant comorbidities, shorter life expectancy, and other favorable disease characteristics (PSA, MRI). Exclusion criteria for AHS include men with serum PSA values >10, greater than 4 of 12 cores positive for cancer with tumor volume >50% in each core.

### 2.2. Holistic Regimen

Dietary recommendations include eliminating red meats, dairy products, fried foods, and refined carbohydrates from the patient's everyday diet. Our protocol emphasizes consuming poultry, fish, green tea, soy milk, red wine, and flaxseed in place of carcinogenic foods. In addition, patients are encouraged to add more fresh vegetables to their everyday diet, with an emphasis on cruciferous vegetables. AHS protocol replaces all cow milk with soy milk. At each visit, patients were reminded about the protocol and were asked if they were compliant with the diet and supplements as outlined in Table 1.

In addition, AHS patients were taking the following chemopreventive supplements to their diet (Table 1). Our institution has extensive first-hand experience with these particular supplements and thus recommends them to our patients as they have proven to be safe and effective. However, the supplements listed herein may be substituted with other chemopreventive agents that have been proven to elicit the same host response.

### 2.3. Follow-Up

Our Active Holistic follow-up protocol includes a PSA every three months, digital rectal examination, and an annual mpMRI scan. If PSA doubling time (PSADT) was less than 12 months, repeat PSA testing was required to ensure accuracy. Criteria for progression on mpMRI includes extracapsular extension, development of a new focus of tumor, or an enlargement of an already existing tumor. Rise in PSA (PSADT < 12 months), unfavorable genomics result, and progression on mpMRI all warrant a biopsy confirmation or an opportunity for definitive treatment, depending on patient preference. If a confirmation biopsy is performed, Gleason upgrading to (3 + 4 or 4 + 3) and an increase of volume of the original tumor (involvement of more cores) are all indications for definitive treatment. Patient preference was an additional indication for obtaining definitive treatment.

### 2.4. Statistical Methods

Demographic data was obtained including: median age, follow-up, and initial PSA. Patients were stratified by Gleason score into Gleason 6 (3 + 3), Gleason 7 (3 + 4), and Gleason 7 (4 + 3). Average PSA over time for the cohort was calculated. The primary outcomes were overall and cancer specific survival and cessation of surveillance and initiation of definitive treatment. Kaplan-Meier estimates were generated using R-Project. The secondary outcomes included the reason for discontinuation and definitive treatments following AHS.

## 3. Results

**Figure 1:**
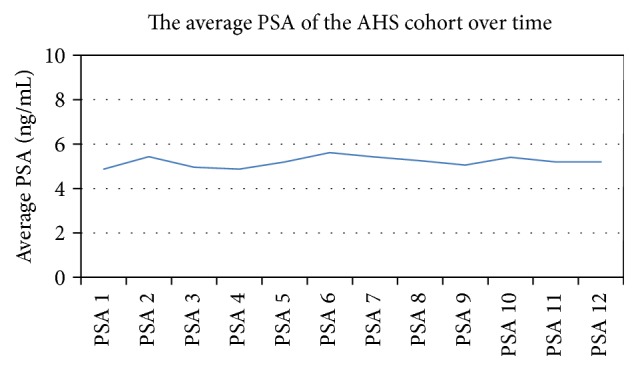
The average PSA of the total cohort while on AHS.

235 patients met inclusion criteria. The distribution of age, follow-up, pre-Gleason score, and initial PSA are summarized in Table 2. Median age and follow-up for the cohort were 66 (44–88) years and 42 (3–166) months, respectively. On initial biopsy prior to AHS, 178 patients (76%) were found to have a Gleason score of 6 (3 + 3). 35 (14.9%) patients had a pre-AHS biopsy with Gleason score of 7 with 29 (12.3%) Gleason 3 + 4 and 6 (2.6%) having a Gleason score of 4 + 3, respectively. The median baseline PSA for patients enrolled in AHS was 4.1 ng/mL.  Figure 1 shows the change in average PSA for the AHS cohort during surveillance.

**Figure 2:**
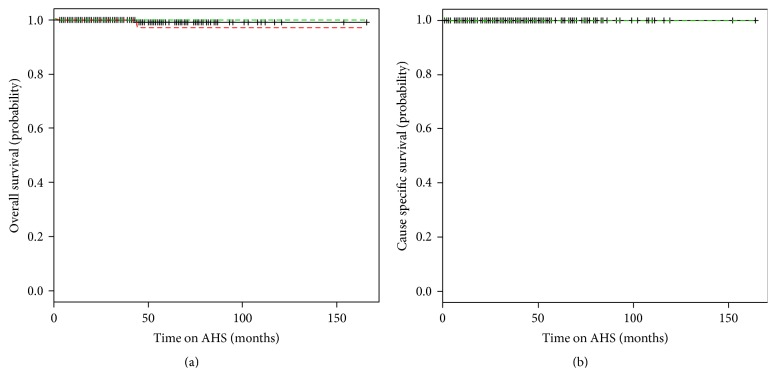
Kaplan-Meier survival curves. (a) The overall survival of the AHS cohort. (b) The disease specific survival of the AHS cohort (key: solid black line represents the probability; red and green lines represent the standard deviation of the curve).

The overall and prostate cancer specific survival for the cohort was 99.6% and 100% at a median follow-up of 42 months (range 3–166) (Figure 2).

**Figure 3:**
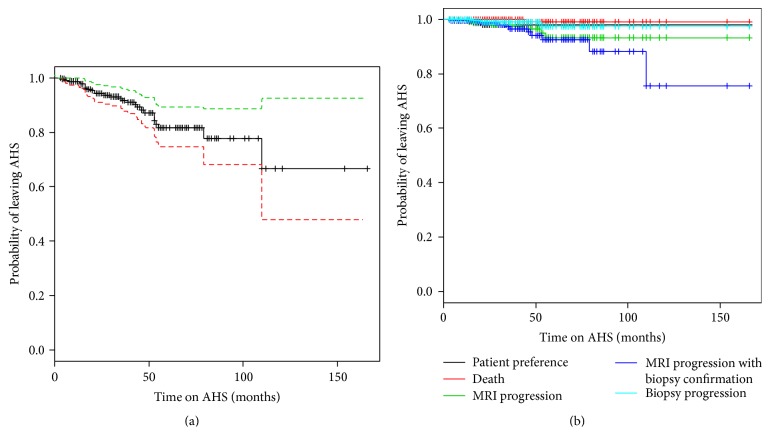
Kaplan-Meier survival curves. (a) The overall likelihood of patients remaining on AHS cohort (key: solid black line represents the probability; red and green lines represent the standard deviation of the curve). (b) The likelihood of patients remaining on AHS stratified by the reasons for intervention.

At last analysis (December 2015), 27 patients (11.5%) received definitive treatment.  Table 3 summarizes the rate of discontinuing AHS per year. The overall dropout rate was 11.5%. The probability of remaining on AHS was 94%, 82%, and 67%, at 2 years, 5 years, and 10 years, respectively (Figure 3(a)).

Table 4 evaluates the 27 patients who discontinued AHS. The median age of these men was higher than the overall cohort (70 years). 26% of these patients had a Gleason of 7 (both 3 + 4 and 4 + 3) compared to only 16% of the overall cohort. The initial median PSA, 5 ng/mL, was also higher than the overall cohort's. The reasons that patients discontinued AHS are found in Table 5. MRI progression with biopsy confirmation was the most common reason (40.47%). MRI progression without a biopsy and biopsy progression without an MRI were the next likely reasons patients discontinued AHS with 14.81% and 11.11%. Three patients (1.3%) left the protocol because of preferences which usually was a result of patient anxiety and/or fluctuations in PSA values. One patient died of an unrelated illness. A Kaplan-Meier curve illustrates the probability of patients to continue AHS stratified by these reasons shown on Figure 3(b).

Of the patients who opted for definitive intervention 58% had cryotherapy, 30% had CyberKnife, and 1 patient had a radical prostatectomy, shown in Table 6. In addition, 1 patient elected for androgen deprivation therapy.

## 4. Discussion

Today, the number of patients who are choosing to go on active surveillance rather than undergo definitive treatment is growing. In addition, there is increasing physicians' support of actively watching patients. As a result, numerous physicians and centers across the country have developed an active surveillance regimen. The AHS regimen differs greatly from other active surveillance protocols in the implementation of lifestyle changes, including the alteration of diet and the addition of supplements and physical activity. The dietary portion of the protocol adopts an anti-inflammatory, antioxidant chemopreventive approach where the patient reduces the factors that may contribute to chronic inflammation. Reduction of saturated and trans fats found in animal foods is implemented in the diet in order to reduce the risk of prostate cancer. Other bases of the holistic protocol are the addition of soy, flaxseed, AHCC, vitamin D3, and Omega 3 Fish Oil.

The ideal candidate for AHS at our institution matches that of many other active surveillance protocols that is characterized by a patient with low to intermediate prostate cancer. While early literature shown active surveillance to be promising for Gleason ≤6, recent studies have broadened the inclusion criteria [32]. In an active surveillance study performed at the University of Toronto, the cohort had successful overall outcomes with 25% of the patients classified as intermediate risk and the remaining patients had low risk characteristics [4]. The majority of the AHS patients, 76%, would be classified as low risk according to the D'Amico risk groups, and the 24% would be considered intermediate risk. These numbers are comparable to the risk distribution at other institutions.

It has been reported that active surveillance is safe and nonlethal at duration of up to 15 years [4]. In a long term study of an active surveillance cohort of 993 patients with a median follow-up of 6.4 years (with a range of 0.2–19.8 years), the overall survival in the cohort was 80% at 10 years [32]. The overall survival of the AHS cohort is comparable if not better than the literature values at 99%. Similarly, the cause specific survival reported in previous literature has ranged from 0% to 1.8% [4, 32]. In the AHS cohort, no patients have died from prostate cancer. Thus, this study supports the results of previous studies in that low to intermediate risk prostate cancer patients are the ideal candidates for active surveillance.

Overall, 11% of AHS patients went on to receive definitive treatment with a median follow-up of 3.5 years (with a range of 0.3–13.8 years). This is lower than both the Toronto and Hopkins groups, where 27% and 33.3% of their patients have been treated definitively with a median follow-up of 6.4 and 6.5 years [4, 32]. Despite the fact that these studies have a longer follow-up, our average dropout rate per year, 2.35%, is lower than the 8.8% rate previously reported [33]. Recent analysis from Klotz et al. published that 75.7%, 63.5%, and 55.0% of patients remained untreated and on surveillance at 5, 10, and 15 years, respectively [4]. Another study performed at the University of California San Francisco showed 2- and 5-year continuation on active surveillance to be 85% and 67%, respectively [34]. The AHS 2-year, 5-year, and 10-year continuation rates are 94%, 82%, and 67%, respectively. Thus, with 3.5-year follow-up, patients in the AHS cohort are more likely to remain on surveillance than previously reported.

There are limitations to this study. One is that there are many factors that go into AHS including patient selection, lifestyle changes, and means of follow-up. This makes it difficult to measure the impact that diet and lifestyle changes have on the low rates of progression seen in the AHS patients. A univariate and multivariate analysis should be performed in future studies. Furthermore, another major limitation of this study is the inability to measure patients' compliance to these recommended diet and lifestyle changes. Aside from patient confirmation there is no means to measure to what degree they have made these changes. Lastly, long follow-up analysis should be performed on the patients who discontinued AHS to establish rates of recurrence and metastasis.

## 5. Conclusion

In our cohort of patients that we selected for surveillance, the incorporation of evidence-based diet and supplements allowed men to stay on the protocol for many years, preventing the need for definitive therapy. Longer follow-up and perhaps a randomized trial comparing AS to AHS should be done to in the future.

## Figures and Tables

**Table 1:** Active Holistic Surveillance supplements.

Supplement	Directions	Rationale	Citations
BroccoProtect	3 capsules, daily	(i) Rich in glucosinolates and antioxidants (ii) Neutralizes the effect of heterocyclic amines found in meats (iii) Induces the apoptosis of prostate cancer cells and prohibits these cells from proliferating (iv) Protects somatic DNA from carcinogenic damage	[6–8]

Omega 3	2000 mg, daily	(i) Suppresses inflammation (ii) Inhibits growth factor induced proliferation in prostate cancer cells	[9, 10]

Zyflamend	3 capsules, daily	(i) Suppresses inflammation (ii) Reduces growth of prostate cell lines (iii) Induces apoptosis of prostate cancer cells	[11, 12]

Vitamin D3	5000 IU, daily	(i) Promotes differentiation of prostate cancer cells (ii) Induces apoptosis of prostate cancer cells (iii) Attenuates proliferation	[13–15]

Genikinoko (GCP)	1000 mg, twice daily	(i) Rich in genistein (ii) Causes cell cycle growth arrest (iii) Induces apoptosis of prostate cancer cells (iv) Antiangiogenesis properties in vitro and in vivo (v) Reduces serum PSA	[6, 16–19]

Active Hexose Correlated Compound (AHCC)	3 capsules, daily	(i) Boosts host immunity (ii) Protects against disorders induced by oxidative stress (iii) Reduces serum PSA	[20–24]

Lycocell	2 capsules, daily	(i) Lycopene complex (ii) Antioxidants protect DNA from damage (iii) Causes prostate cancer cell cycle arrest and apoptosis (iv) Induces differentiation (v) Reduces oxidative stress	[25, 26]

**Table 2:** Patient demographics (*N* = 235).

Age, median (range), years	66 (44–88)
Time on AHS, median (range), months	42 (3–166)
Gleason score	
6	178
7 (3 + 4)	29
7 (4 + 3)	6
N/R	22
Initial PSA, median (range), ng/mL	4.1 (0.5–15.6)

**Table 3:** The discontinuation rate for patients on AHS.

Year	Started AHS (*n*)	Total on AHS	Off AHS (*n*)	Rate off AHS
Before 2010	32	32	0	0.00%
2010	25	57	0	0.00%
2011	30	87	0	0.00%
2012	61	148	0	0.00%
2013	35	175	8	4.57%
2014	36	199	12	6.03%
2015	16	209	7	3.35%
Overall	235		27	11.50%

**Table 4:** Demographics of patients who discontinued AHS (*N* = 27).

Age, median (range), years	70 (57–80)
Time on AHS, median (range), months	30 (4–110)
Gleason score	
6	18
7 (3 + 4)	6
7 (4 + 3)	1
N/R	2
Initial PSA, median (range), ng/mL	5 (0.8–15.6)

**Table 5:** Reason for intervention on AHS.

Reasons for discontinuing AHS	*N* (%)
Biopsy progression	4 (14.81)
MRI progression	8 (29.63)
MRI progression confirmed with biopsy	11 (40.74)
Patient preference	3 (11.11)
Deceased	1 (3.70)
Total	27 (100)

**Table 6:** Treatment options of the patients that discontinued (*N* = 26).

Cryotherapy	15
CyberKnife	8
Radical prostatectomy	1
ADT	1
N/A	1
